# Sum Rate Optimization of IRS-Aided Uplink Muliantenna NOMA with Practical Reflection

**DOI:** 10.3390/s22124449

**Published:** 2022-06-12

**Authors:** Jihyun Choi, Luiggi Cantos, Jinho Choi, Yun Hee Kim

**Affiliations:** 1 Department of Electronic Engineering, Kyung Hee University, 1732 Deogyeong-daero, Giheung-gu, Yongin-si 17104, Gyeonggi-do, Korea; solbangwool_@khu.ac.kr; 2 Department of Electronics and Information Convergence Engineering, Kyung Hee University, 1732 Deogyeong-daero, Giheung-gu, Yongin-si 17104, Gyeonggi-do, Korea; lrcantos@khu.ac.kr; 3 School of Information Technology, Deakin University, Burwood, VIC 3125, Australia; jinho.choi@deakin.edu.au

**Keywords:** intelligent reflecting surface, nonorthogonal multiple access, practical reflection, multiple receive antennas

## Abstract

Recently, intelligent reflecting surfaces (IRSs) have drawn huge attention as a promising solution for 6G networks to enhance diverse performance metrics in a cost-effective way. For massive connectivity toward a higher spectral efficiency, we address an intelligent reflecting surface (IRS) to an uplink nonorthogonal multiple access (NOMA) network supported by a multiantenna receiver. We maximize the sum rate of the IRS-aided NOMA network by optimizing the IRS reflection pattern under unit modulus and practical reflection. For a moderate-sized IRS, we obtain an upper bound on the optimal sum rate by solving a determinant maximization (max-det) problem after rank relaxation, which also leads to a feasible solution through Gaussian randomization. For a large number of IRS elements, we apply the iterative algorithms relying on the gradient, such as Broyden–Fletcher–Goldfarb–Shanno (BFGS) and limited-memory BFGS algorithms for which the gradient of the sum rate is derived in a computationally efficient form. The results show that the max-det approach provides a near-optimal performance under unit modulus reflection, while the gradient-based iterative algorithms exhibit merits in performance and complexity for a large-sized IRS with practical reflection.

## 1. Introduction

Intelligent reflecting surfaces (IRSs) have drawn enormous attention from the academy and industry as a cost-effective building block for 6G wireless communication networks demanding high spectral and energy efficiency [1,2,3]. An IRS constructed with a large number of passive reflection elements can reconfigure a wireless propagation channel to be favorable for information or energy transfer by controlling their reflecting patterns. In doing so, the IRS avoids a large power consumption with passive elements and achieves a full-duplex gain without complicated signal processing such as interference cancellation and demodulation. In this aspect, various IRS-assisted wireless communications have been explored for their own purposes, from basic multiuser or/and multiantenna communication systems [4,5,6,7,8,9] to more complicated system configurations, as surveyed in Ref. [10].

For multiuser communications, nonorthogonal multiple access (NOMA) craving for higher spectral efficiency and massive connectivity has been considered as a promising candidate for 6G networks [11,12,13,14,15,16]. Hence, many recent IRS studies have been devoted to IRS-assisted NOMA for further improvement in spectral efficiency, energy efficiency, and reliability [17,18,19,20,21,22,23,24,25,26,27]. One of the major concerns of IRS-aided NOMA networks resides in how to reflect a superimposed NOMA signal appropriately to meet the design goal of a given network. In the downlink, joint optimization of IRS reflection and power allocation was studied with a single-antenna base station (BS) to minimize the transmit power of a two-user NOMA signal [18] and maximize the sum rate of a multiuser NOMA signal [19]. For a multiantenna BS in the downlink, transmit beamforming and IRS reflection were optimized without or with power allocation to maximize the sum rate [20], minimize the transmit power [21,22,23], and maximize the minimum rate [24]. On the other hand, in the uplink, IRS-aided NOMA was studied to maximize the sum rate achieved with a single-antenna receiver by optimizing the IRS reflection vector [27]. The sum rate maximization problem was also extended to IRS-aided NOMA accompanied by wireless power transfer with a single-antenna receiver [28,29] and a multiantenna receiver [30].

The recent studies on IRS-aided NOMA networks have assumed unit modulus reflection for IRS since the phase control is more readily implementable than the amplitude control in implementing passive IRS elements. It should also be noted that IRS optimization under unit modulus reflection has resorted to semidefinite relaxation (SDR) solving a semidefinite program (SDP) [31] for the downlink with a given transmit beamforming and for the uplink with a single-antenna BS, where the SDP deals with linear functions of semidefinite matrices [20,23,24,27,28,29]. However, the SDR approach is less favorable for an IRS requiring a large number of passive elements to compensate for double fading due to its complexity increasing polynomially with the number of elements. To reduce the complexity of the SDR, an iterative algorithm based on a second-order surrogate function similar to the gradient descent was proposed in Ref. [5] for the simple objective function given by the trace of a matrix.

For the uplink NOMA with a multiantenna BS [30], the sum rate is given in a more complicated log-determinant expression similar to a multiple-input multiple-output (MIMO) capacity [7]. In this case, the SDP, having linear functions of semidefinite matrices in the objective and constraints, is no longer applicable. A few studies have dealt with such a complicated objective function in IRS reflection optimization [7,30], which was optimized through a suboptimal sequential optimization method, optimizing one IRS element while fixing the other elements without knowing its performance gap to the optimal one. In addition, practical IRS reflection models with phase-dependent amplitudes observed in practical circuits [32] have not been studied for IRS-aided NOMA networks yet. In this regard, we consider a sum rate maximization problem of an IRS-aided uplink NOMA network with a multiantenna BS and tackle the problem under a generalized IRS reflection model encompassing unit modulus and practical reflection [32]. The main contributions are summarized as follows:

- We formulate the sum rate optimization problem by incorporating the optimal receive beamforming for a given IRS phase vector in the objective function, which is characterized by the log-determinant of a matrix as in the MIMO capacity expression [7,30]. As a result, only the IRS phase vector needs to be optimized in the uplink, while the transmit beamforming and IRS phase vectors have been optimized alternately in the downlink [5];
- For a moderate-sized IRS with unit modulus reflection, we propose an extended SDR approach that converts the sum rate maximization problem into a determinant maximization (max-det) problem [33]. The max-det solution not only provides an upper bound on the optimal sum rate of the IRS-aided uplink multiantenna NOMA, but also leads to a rank-one feasible solution resulting in a near-optimal performance. This approach can also be employed to obtain an upper bound on the IRS-aided MIMO capacity under unit modulus reflection;
- For a large-sized IRS under generalized reflection, we reformulate the problem as an unconstrained nonlinear optimization problem that can be solved by using gradient-based iterative algorithms [34]. In particular, we use the more sophisticated Broyden–Fletcher–Goldfarb–Shanno (BFGS) and limited-memory BFGS (L-BFGS) algorithms [34,35], while the gradient-descent approach is used in [5]. For an efficient implementation of such iterative algorithms, we derive the gradient of the complicated objective function in a computationally efficient form under the generalized reflection;
- We analyze the computational complexity of the iterative algorithms when the derived gradient is used to update the search point. The results show that the iterative algorithms reduce the complexity of the extended SDR with max-det optimization significantly. In addition, the iterative algorithms provide a performance gain over the conventional sequential optimization method [7] at a reduced computational time.

*Notation*: The sets of n×m complex-valued and real-valued matrices are denoted by $C^{n\times m}$ and $R^{n\times m}$, respectively, with $C^{n}=C^{n\times 1}$ and $R^{n}=R^{n\times 1}$, while the set of n×n positive semidefinite Hermitian matrices is denoted by $S_{+}^{n}$. The transpose, Hermitian, and trace are denoted by ${\left(\cdot \right)}^{T}$, ${\left(\cdot \right)}^{H}$, and tr(·), respectively. We use diag(a) for the diagonal matrix with a diagonal vector a, ${\left[a\right]}_{n}$ for the *n*th entry of a vector a, and, ${\left[A\right]}_{n,m}$ for the (n,m)th entry of a matrix A, and CN(μ,Σ) for complex Gaussian distribution with mean vector μ and covariance matrix Σ.

## 2. System Model and Problem Formulation

### 2.1. System Model

**Figure 1:**
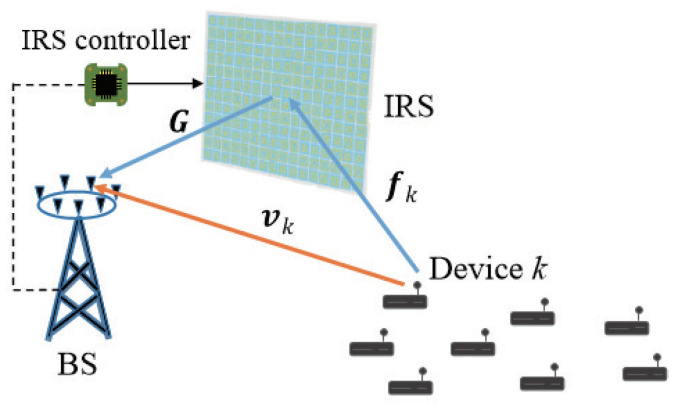
IRS-aided uplink consisting of a multiantenna BS, *K* single-antenna devices, and an IRS with *N* elements.

We consider the uplink of a single-cell network described in Figure 1. The network consists of a BS equipped with *M* antennas, *K* devices equipped with a single antenna, and an IRS comprising *N* reflection elements. The channels from device *k* to the BS and to the IRS are denoted by $v_{k}\in C^{M}$ and $f_{k}\in C^{N}$, respectively, for k∈K≜{1,2,…,K}. The channel from the IRS to the BS is denoted by $G\in C^{M\times N}$. The IRS reflection vector is denoted by $\theta ={\left[\theta_{1},\theta_{2},\ldots ,\theta_{N}\right]}^{T}\in C^{N}$, where $\theta_{n}=e^{j\phi_{n}}$ for n∈N≜{1,2,…,N} under unit modulus reflection. To address the amplitude distortion of practical IRS control circuits depending on the phase, we express the IRS reflection in a generalized form as [32]

(1) $$\begin{array}{c} \theta_{n}=\beta \left(\phi_{n}\right)e^{j\phi_{n}},\;n\in N, \end{array}$$

where

(2) $$\begin{array}{c} \beta_{n}\left(\phi_{n}\right)=\left(1-\beta_{\min}\right){\left(\frac{\sin(\phi_{n}-\phi_{0})+1}{2}\right)}^{\alpha }+\beta_{\min} \end{array}$$

with α≥0, $\beta_{\min}\geq 0$, and $\phi_{0}\geq 0$. The values for parameters α, $\beta_{\min}$, and ϕ in (2) are determined by the specific circuit implementation, where (2) with α=0 represents the unit modulus reflection with $\beta_{n}\left(\phi_{n}\right)=1$.

For the uplink transmission, we allow *K* devices to transmit their symbols simultaneously, where the number *K* of devices is larger than the number *M* of receiving antennas for NOMA. However, the following results are also applicable to space division multiple access with K≤M. The signal received at the BS is then written as

(3) $$\begin{array}{c} y=\sum_{k=1}^{K}\sqrt{p_{k}}\left(G\mathrm{diag}\left(\theta \right)f_{k}+v_{k}\right)s_{k}+z, \end{array}$$

where $s_{k}$ and $p_{k}$ are the symbol and transmit power of device k∈K, respectively, and $z∼\mathrm{CN}(0,\sigma^{2}I_{M})$ is the noise vector added at the BS. We can express the received signal (3) in a concise form as

(4) $$\begin{array}{c} y=\sum_{k=1}^{K}\sqrt{p_{k}}H_{k}\tilde{\theta }s_{k}+z, \end{array}$$

where $H_{k}$ is the equivalent channel from device *k* to the BS, which is given by

(5) $$\begin{array}{c} H_{k}=\left[G\mathrm{diag}\left(f_{k}\right)\;v_{k}\right]\in C^{M\times (N+1)}, \end{array}$$

and $\tilde{\theta }={\left[\theta^{T}\;1\right]}^{T}\in C^{N+1}$ is the extended IRS reflection vector.

Without loss of generality, we assume that the devices are indexed in the successive interference cancellation (SIC) order. The BS detects the device symbols from $s_{1}$ to $s_{K}$ sequentially by applying receive beamforming $w_{k}\in C^{M}$ to the received signal after SIC in detecting $s_{k}$, specifically by applying the receive beamforming $w_{k}$ to the received signal after $\left\{s_{1},s_{2},\ldots ,s_{k-1}\right\}$ being detected and canceled, which is given by

(6) $$\begin{array}{c} {\hat{y}}_{k}=y-\sum_{l=1}^{k-1}\sqrt{p_{l}}H_{l}\tilde{\theta }s_{l}=\sum_{l=k}^{K}\sqrt{p_{l}}H_{l}\tilde{\theta }s_{l}+z, \end{array}$$

where ${\hat{y}}_{1}=y$. We obtain

(7) $$\begin{array}{c} {\tilde{y}}_{k}=w_{k}^{H}{\hat{y}}_{k}=\sum_{l=k}^{K}\sqrt{p_{l}}w_{k}^{H}H_{l}\tilde{\theta }s_{l}+w_{k}^{H}z \end{array}$$

from which $s_{k}$ can be detected. Thus, the signal-to-interference-and-noise ratio (SINR) in detecting $s_{k}$ from ${\tilde{y}}_{k}$ is given by

(8) $$\begin{array}{c} \gamma_{k}=\frac{p_{k}{\left|w_{k}^{H}H_{k}\tilde{\theta }\right|}^{2}}{\sum_{l=k+1}^{K}p_{l}{\left|w_{k}^{H}H_{l}\tilde{\theta }\right|}^{2}+\sigma^{2}{∥w_{k}∥}^{2}}. \end{array}$$

The optimal receive beamforming that maximizes the SINR is given by the minimum mean square error (MMSE) beamforming, expressed as [36]

(9) $$\begin{array}{c} w_{k}^{o}=B_{k+1}^{-1}H_{k}\tilde{\theta } \end{array}$$

with

(10) $$\begin{array}{c} B_{k}=\sigma^{2}I_{M}+\sum_{l=k}^{K}p_{l}H_{l}\tilde{\theta }{\tilde{\theta }}^{H}H_{l}^{H} \end{array}$$

for k∈K and $B_{K+1}=\sigma^{2}I_{M}$. The maximum SINR of device *k* achieved with the optimal beamforming $w_{k}^{o}$ is given by

(11) $$\begin{array}{c} \gamma_{k}^{o}=p_{k}{\tilde{\theta }}^{H}H_{k}^{H}B_{k+1}^{-1}H_{k}\tilde{\theta } \end{array}$$

which leads to the achievable rate as follows:

(12) $$\begin{array}{c} R_{k}=\log_{2}\left(1+\gamma_{k}^{o}\right)=\log_{2}\det\left(B_{k}\right)-\log_{2}\det\left(B_{k+1}\right), \end{array}$$

using the matrix determinant lemma, $\det\left(B+uu^{H}\right)=\det\left(B\right)\det\left(1+u^{H}B^{-1}u\right)$ for an invertible matrix B [15,36], and $B_{k}=B_{k+1}+p_{k}H_{k}\tilde{\theta }{\tilde{\theta }}^{H}H_{k}^{H}$. Letting $B=B_{k+1}$ and $u=\sqrt{p_{k}}H_{k}\tilde{\theta }$, we have $\det\left(B_{k}\right)=\det\left(B_{k+1}\right)\left(1+p_{k}{\tilde{\theta }}^{H}H_{k}^{H}B_{k+1}^{-1}H_{k}\tilde{\theta }\right)$, which leads to (12). From (12), the sum rate of all devices is given by Refs. [15,36]

(13) $$\begin{array}{c} R_{\mathrm{sum}}=\sum_{k=1}^{K}\log_{2}\left(1+\gamma_{k}^{o}\right)=\log_{2}\det\left(B_{1}\right)-\log_{2}\det\left(B_{K+1}\right), \end{array}$$

where $B_{1}=\sigma^{2}I_{M}+\sum_{l=1}^{K}p_{l}H_{l}\tilde{\theta }{\tilde{\theta }}^{H}H_{l}^{H}$ and $B_{K+1}=\sigma^{2}I_{M}$ are irrelevant to the SIC order. Note that device rates in (8) depend on the SIC order since the SIC order affects $B_{k}$ for 2≤k≤K. However, the sum rate (13) determined by $B_{1}$ and $B_{K+1}$ does not depend on the SIC order. Finally, the sum rate is expressed as a function of transmit power $p={\left[p_{1},p_{2},\ldots ,p_{K}\right]}^{T}$ of the devices and IRS phase shifts $\phi ={\left[\phi_{1},\phi_{2},\ldots ,\phi_{N}\right]}^{T}$, i.e.,

(14) $$\begin{array}{c} R_{\mathrm{sum}}\left(p,\phi \right)=\log_{2}\det\left(I_{M}+\sum_{k=1}^{K}\frac{p_{k}}{\sigma^{2}}H_{k}\tilde{\theta }{\tilde{\theta }}^{H}H_{k}^{H}\right), \end{array}$$

where $\tilde{\theta }$ is a function of ϕ.

### 2.2. Problem Formulation

This paper aims to maximize the sum rate of the IRS-aided uplink NOMA by optimizing the transmit power $p={\left[p_{1},p_{2},\ldots ,p_{K}\right]}^{T}$ of the devices and the phase shifts $\phi ={\left[\phi_{1},\phi_{2},\ldots ,\phi_{N}\right]}^{T}$ of the IRS as follows:

(15a) $$\begin{array}{ccc} \; & \max_{p\in R^{K},\phi \in R^{N}} & R_{\mathrm{sum}}\left(p,\phi \right) \end{array}$$

(15b) $$\begin{array}{ccc} \; & s.t.\; & 0\leq p_{k}\leq P_{k},\;k\in K, \end{array}$$

(15c) $$\begin{array}{ccc} \; & & 0\leq \phi_{n}\leq 2\pi ,\;n\in N. \end{array}$$

Since the sum rate (14) is a non-decreasing function of $p_{k}$ irrespective of ϕ, the optimal power of problem (15) is given by

(16) $$\begin{array}{c} p^{o}=P≜{\left[P_{1},P_{2},\ldots ,P_{K}\right]}^{H} \end{array}$$

that results in the sum rate as

(17) $$\begin{array}{c} R\left(\phi \right)≜R_{\mathrm{sum}}\left(P,\phi \right)=\log_{2}\det\left(I_{M}+S\left(\phi \right)\right) \end{array}$$

with

(18) $$\begin{array}{c} S\left(\phi \right)=\sum_{k=1}^{K}\xi_{k}H_{k}\tilde{\theta }{\tilde{\theta }}^{H}H_{k}^{H} \end{array}$$

and $\xi_{k}=P_{k}/\sigma^{2}$.

Finally, the sum rate optimization problem (15) becomes

(19a) $$\begin{array}{ccc} & \max_{\phi \in R^{N}} & R(\phi ) \end{array}$$

(19b) $$\begin{array}{ccc} & s.t.\; & 0\leq \phi_{n}\leq 2\pi ,\;\forall n. \end{array}$$

It should be noted that problem (19) under unit modulus reflection is equivalent to a subproblem of the IRS-aided MIMO capacity optimization problem [7], which was solved by the customized sequential optimization method. In the following, we will provide alternative methods providing either a better performance under unit modulus reflection or a faster computation under generalized reflection than the conventional method [7].

## 3. IRS Reflection Optimization

### 3.1. Determinant Maximization for a Moderate-Sized IRS

This subsection tackles the problem (19) for a moderate *N* by extending the SDR approach. For this purpose, let us rewrite the signal matrix (18) as

(20) $$\begin{array}{c} S\left(\phi \right)=H\left(\left(\tilde{\theta }{\tilde{\theta }}^{H}\right)⊗\Xi \right)H^{H}, \end{array}$$

where $\Xi =\mathrm{diag}({\left[\xi_{1},\xi_{2},\ldots ,\xi_{K}\right]}^{T})$, $H=\left[H_{1},H_{2},\ldots ,H_{K}\right]\in C^{M\times K(N+1)}$, and ⊗ denotes the Kronecker product. The signal matrix is a Hermitian semidefinite matrix and is linear with $\tilde{X}$ when $\tilde{X}=\tilde{\theta }{\tilde{\theta }}^{H}$. We define $\tilde{X}=\tilde{\theta }{\tilde{\theta }}^{H}\in S_{+}^{N+1}$, of which the diagonal entries satisfy ${\tilde{X}}_{n,n}={\left|{\tilde{\theta }}_{n}\right|}^{2}\leq 1$. In this case, we can transform (19) into

(21a) $$\begin{array}{ccc} & \;\max_{\tilde{X}\in S_{+}^{N+1}} & \tilde{R}\left(\tilde{X}\right)≜\log_{2}\det\left(I_{M}+H\left(\tilde{X}⊗\Xi \right)H^{H}\right) \end{array}$$

(21b) $$\begin{array}{ccc} & \;s.t.\; & {\tilde{X}}_{n,n}\leq 1,\;n=1,2,\ldots ,N+1, \end{array}$$

(21c) $$\begin{array}{ccc} & & \mathrm{rank}(\tilde{X})=1. \end{array}$$

Let ${\tilde{X}}_{o}$ and ${\tilde{R}}_{o}=\tilde{R}\left({\tilde{X}}_{o}\right)$ denote the optimal solution of (21) and the corresponding optimal sum rate, respectively. Since finding ${\tilde{X}}_{o}$ is intractable due to the rank constraint, we resort to an approximate solution by finding a rank-relaxed solution first and then estimating a rank-one solution from the rank-relaxed one as follows.

In the first step, by relaxing the rank constraint (21c), we can approximate (21) to the max-det problem defined in [33], which is expressed in the standard from as follows:

(22a) $$\begin{array}{ccc} & \;\min_{\tilde{X}\in S_{+}^{N+1}} & \;\log_{2}\det\left(Y^{-1}\right) \end{array}$$

(22b) $$\begin{array}{ccc} & \;s.t.\; & \;Y≜I_{M}+H\left(\tilde{X}⊗\Xi \right)H^{H}≻0, \end{array}$$

(22c) $$\begin{array}{ccc} & \; & \;F≜\mathrm{diag}(1-{\tilde{X}}_{1,1},\ldots ,1-{\tilde{X}}_{N+1,N+1})⪰0. \end{array}$$

Since (22) is known to be a convex optimization problem with linear matrix inequalities in (22b) and (22c), it can be solved with an existing convex optimization solver, which leads to the optimal solution ${\tilde{X}}_{⋄}$ and ${\tilde{R}}_{⋄}=\tilde{R}\left({\tilde{X}}_{⋄}\right)$. Here, the optimal value ${\tilde{R}}_{⋄}$ of the rank-relaxed problem (22) can serve as an upper bound on the optimal value ${\tilde{R}}_{o}$ of the rank-constrained problem (21), since the constraint set of (22) includes that of (21).

In the second step, from the solution ${\tilde{X}}_{⋄}$ of (22), we obtain a rank-one solution close to ${\tilde{X}}_{o}={\tilde{\theta }}_{o}{\tilde{\theta }}_{o}^{H}$ though the Gaussian randomization procedure [24,31]. Since any rank-one solution $\tilde{X}$ of (21) is decomposed as $\tilde{X}=\tilde{x}{\tilde{x}}^{H}$, the Gaussian randomization procedure generates *L* zero-mean complex Gaussian samples as ${\left\{{\tilde{x}}_{l}∼\mathrm{CN}\left(0,{\tilde{X}}_{⋄}\right)\right\}}_{l=1}^{L}$ for a rank-one solution so that ${\tilde{x}}_{l}{\tilde{x}}_{l}^{H}$ resembles ${\tilde{X}}_{⋄}$ as $E\left[{\tilde{x}}_{l}{\tilde{x}}_{l}^{H}\right]={\tilde{X}}_{⋄}$. We then obtain ${\tilde{\tilde{x}}}_{l}=e^{-j∠{\left[{\tilde{x}}_{l}\right]}_{N+1}}{\tilde{x}}_{l}$ to align the phases with ${\tilde{\theta }}_{o}={\left[\theta_{o}^{T}\;1\right]}^{T}$, where *∠* denotes the phase of a complex number. Note that the statistics remain unchanged by the phase shift since ${\tilde{\tilde{x}}}_{l}{\tilde{\tilde{x}}}_{l}^{H}={\tilde{x}}_{l}{\tilde{x}}_{l}^{H}$. We then obtain a feasible candidate ${\tilde{X}}_{l}$ subject to ${\left[{\tilde{X}}_{l}\right]}_{n,n}\leq 1$ for the solution of (21), which is equivalent to obtaining ${\tilde{\theta }}_{l}={\left[\theta_{l}^{T}\;1\right]}^{T}$ subject to ${\left[\theta_{l}\right]}_{n}=\beta \left({\left[\phi_{l}\right]}_{n}\right)e^{j{\left[\phi_{l}\right]}_{n}}$ for n∈N. To generate ${\tilde{\theta }}_{l}$, we obtain the phases as ${\left[\phi_{l}\right]}_{n}=∠{\left[{\tilde{\tilde{x}}}_{l}\right]}_{n}$ for n∈N. The phase vectors ${\left\{\phi_{l}\right\}}_{l=1}^{L}$ generated by the phases of complex numbers are feasible for problem (19) and ${\left\{{\tilde{X}}_{l}={\tilde{\theta }}_{l}{\tilde{\theta }}_{l}^{H}\right\}}_{l=1}^{L}$ are also feasible for problem (21) since ${\left[{\tilde{X}}_{l}\right]}_{n,n}\leq 1$ and $\mathrm{rank}({\tilde{X}}_{l})=1$. Among all the feasible candidates ${\left\{\phi_{l}\right\}}_{l=1}^{L}$ (or equivalently ${\left\{{\tilde{X}}_{l}\right\}}_{l=1}^{L}$), the Gaussian randomization procedure finds the best candidate as $l_{☆}=\arg\max_{1\leq l\leq L}R\left(\phi_{l}\right)=\arg\max_{1\leq l\leq L}\tilde{R}\left({\tilde{X}}_{l}\right)$ for its output. The sum rate $\tilde{R}\left({\tilde{X}}_{l_{☆}}\right)$ with the best candidate ${\tilde{X}}_{l_{☆}}$ is a non-decreasing function of the number *L* of random samples so that $\tilde{R}\left({\tilde{X}}_{l_{☆}}\right)$ is likely to move closer to the optimal sum rate $\tilde{R}\left({\tilde{X}}_{o}\right)$ as *L* increases. Later, we will empirically demonstrate that Gaussian randomization with a sufficient *L* provides a good approximate solution close to the optimal value $\tilde{R}\left({\tilde{X}}_{o}\right)$ of our problem, as in the other SDR applications [31].

**Remark** **1.** *The max-det problem (22) consists of X with l=(N+1)(N+2)/2 complex variables, whilst $Y\in S_{+}^{M}$ and $F\in S_{+}^{N+1}$ are in the constraints. Thus, the problem can be solved by an interior-point algorithm with $O(\left(M^{2}+{\left(N+1\right)}^{2}\right)l^{2})$ operations per search point and a worst-case complexity of $O(\sqrt{N+1})$ iterations [33]. In short, the complexity solving (22) is given by $O(N^{6.5})$, which becomes unacceptably large as N increases.*

### 3.2. Gradient-Based Iterative Algorithms for a Large-Sized IRS

This subsection provides a suboptimal approach to solving problems (19) in a faster way for a large *N* by transforming the problem into an equivalent unconstrained nonlinear optimization problem. For this purpose, we remove the phase constraints in (19) as

(23) $$\begin{array}{c} \max_{\phi \in R^{N}}\;R\left(\phi \right), \end{array}$$

which has the same optimal value with (19) due to the periodicity of R(ϕ) for all the entries of ϕ with period 2π. For ease of exposition, we convert (23) to a minimization problem as

(24) $$\begin{array}{c} \min_{\phi \in R^{N}}\;f\left(\phi \right) \end{array}$$

by defining f(ϕ)=−R(ϕ). Due to the nonconvexity of R(ϕ) and its complicated form, it is almost impossible to obtain the optimal solution of (24) even for unit modulus reflection. Instead of the sequential optimization optimizing one IRS element at a time [7,30], we solve the problem through an iterative algorithm minimizing a local approximation (or a surrogate function) at each iteration to update its IRS phases $\phi_{t}$ for t=0,1,… simultaneously [34]. To accommodate a large *N*, we adopt the algorithms based on second-order Taylor series approximations but relying on the gradient $\nabla f\left(\phi \right)={\left[\frac{\partial f}{\partial \phi_{1}},\frac{\partial f}{\partial \phi_{2}},\ldots ,\frac{\partial f}{\partial \phi_{N}}\right]}^{T}$ in their implementation. The algorithms include the gradient descent (GD) [5,34] and quasi Newton methods such as BFGS and L-BFGS [34,35], which are briefly summarized in the following.

The algorithms are based on second-order Taylor approximations that can be expressed in a generic form as

(25) $$\begin{array}{c} f_{A}\left(\phi ,\phi_{t}\right)=f\left(\phi_{t}\right)+g_{t}^{T}\Delta \phi_{t}+\frac{1}{2}\Delta \phi_{t}^{T}A_{t}\Delta \phi_{t}, \end{array}$$

where $\Delta \phi_{t}=\phi -\phi_{t}$, $g_{t}{=\nabla f\left(\phi \right)|}_{\phi =\phi_{t}}$, and $A_{t}\in C^{N\times N}$ is chosen by an algorithm. The solution is updated by minimizing $f_{A}\left(\phi ,\phi_{t}\right)$ as

(26) $$\begin{array}{c} \phi_{t+1}=\phi_{t}-A_{t}^{-1}g_{t}. \end{array}$$

The GD with the update rule

(27) $$\begin{array}{c} \phi_{t+1}=\phi_{t}-\delta_{t}g_{t} \end{array}$$

for $\delta_{t}>0$ at complexity O(N) is obtained with a choice of $A_{t}=\frac{1}{\delta_{t}}I_{M}$, where the step size $\delta_{t}$ is determined by the Armijo rule [5]. The BFGS and L-BFGS update the search point as

(28) $$\begin{array}{c} \phi_{t+1}=\phi_{t}-\delta_{t}Q_{t}g_{t}, \end{array}$$

where $Q_{t}$ is an estimate of the inverse Hessian $A_{t}^{-1}$ with $A_{t}=\nabla^{2}{f\left(\phi \right)|}_{\phi =\phi_{t}}$ to reduce the complexity of the Newton method computing the Hessian and its inverse. The BFGS estimates $Q_{t+1}$ with $Q_{t}$, $u_{t}=\phi_{t+1}-\phi_{t}$, and $r_{t}=g_{t+1}-g_{t}$ at complexity $O(N^{2})$ [34] whilst the L-BFGS having $m_{B}$ memories estimates $Q_{t+1}$ with ${\left\{u_{i},r_{i}\right\}}_{i=t-m_{B}+1}^{t}$ at complexity $O(m_{B}N)$ for [35].

For the efficient implementation of the aforementioned algorithms, we now derive the gradient of the sum rate with respect to the IRS phase vector $\phi ={\left[\phi_{1},\phi_{2},\ldots ,\phi_{N}\right]}^{T}$, which is denoted by $\nabla R\left(\phi \right)={\left[\frac{\partial R}{\partial \phi_{1}},\frac{\partial R}{\partial \phi_{2}},\ldots ,\frac{\partial R}{\partial \phi_{N}}\right]}^{T}$, in a computationally efficient form for ∇f(ϕ)=−∇R(ϕ). By rewriting

(29) $$\begin{array}{c} R\left(\phi \right)=\frac{1}{\ln2}\ln\det Y\left(\phi \right) \end{array}$$

with $Y\left(\phi \right)=I_{M}+S\left(\phi \right)$, we first obtain

(30) $$\begin{array}{c} \frac{\partial R(\phi )}{\partial \phi_{n}}=\frac{1}{\ln2}\mathrm{tr}\left(Y^{-1}\left(\phi \right)\frac{\partial Y(\phi )}{\partial \phi_{n}}\right)=\frac{1}{\ln2}\mathrm{tr}\left(Y^{-1}\left(\phi \right)\frac{\partial S(\phi )}{\partial \phi_{n}}\right) \end{array}$$

from $\frac{\partial \det(A)}{\partial t}=\det\left(A\right)\mathrm{tr}\left(A^{-1}\frac{\partial A}{\partial t}\right)$. Here, we can compute S(ϕ) in (18) as

(31) $$\begin{array}{ccc} & & \;S\left(\phi \right)=\sum_{n=1}^{N+1}\sum_{l=1}^{N+1}{\tilde{\theta }}_{n}{\tilde{\theta }}_{l}^{*}\sum_{k=1}^{K}\xi_{k}h_{k,n}h_{k,l}^{H}=\tilde{\Theta }H\Xi H^{H}{\tilde{\Theta }}^{H}, \end{array}$$

where $h_{k,n}$ is the *n*th column of $H_{k}$, $\tilde{\Theta }={\tilde{\theta }}^{T}⊗I_{M}$ depends on ϕ as (2), and $H={\left[H_{1}^{T},H_{2}^{T},\ldots ,H_{N+1}^{T}\right]}^{T}$ with $H_{n}=\left[h_{1,n},h_{2,n},\ldots ,h_{K,n}\right]\in C^{M\times K}$. With the signal matrix expressed in (31), we obtain its differentiation as

(32) $$\begin{array}{c} \frac{\partial S(\phi )}{\partial \phi_{n}}=\theta_{n}^{\prime }H_{n}\Xi H^{H}{\tilde{\Theta }}^{H}+{\left(\theta_{n}^{*}\right)}^{\prime }\tilde{\Theta }H\Xi H_{n}^{H}, \end{array}$$

where $\theta_{n}^{\prime }=\frac{d\theta_{n}}{d\phi_{n}}$. For general IRS reflection (2), we have

(33) $$\begin{array}{c} \theta_{n}^{\prime }=\left\{\beta_{n}^{\prime }\left(\phi_{n}\right)+j\beta_{n}\left(\phi_{n}\right)\right\}e^{j\phi_{n}}, \end{array}$$

where

(34) $$\begin{array}{c} \beta_{n}^{\prime }\left(\phi_{n}\right)=\frac{\alpha (1-\beta_{\min})}{2}{\left(\frac{\sin(\phi_{n}-\phi_{0})+1}{2}\right)}^{\alpha -1}\cos\left(\phi_{n}-\phi_{0}\right) \end{array}$$

and ${\left(\theta_{n}^{*}\right)}^{\prime }={\left(\theta_{n}^{\prime }\right)}^{*}$; for unit modulus reflection with α=0, (33) becomes $\theta_{n}^{\prime }=je^{j\phi_{n}}$. With (32), we obtain (30) as

(35) $$\begin{array}{c} \frac{\partial R(\phi )}{\partial \phi_{n}}=\frac{2}{\ln2}ℜ\left\{\theta_{n}^{\prime }\mathrm{tr}\left(H_{n}\Xi H^{H}{\tilde{\Theta }}^{H}Y^{-1}\left(\phi \right)\right)\right\}, \end{array}$$

where ℜ{·} represents the real part and $\Lambda \left(\phi \right)≜\Xi H^{H}{\tilde{\Theta }}^{H}Y^{-1}\left(\phi \right)$ is common to all entries of the gradient. Thus, the gradient is computed as follows:

(36) $$\begin{array}{c} \nabla R\left(\phi \right)=\frac{2}{\ln2}{\left[ℜ\left\{\theta_{1}^{\prime }\mathrm{tr}\left(H_{1}\Lambda \left(\phi \right)\right)\right\},ℜ\left\{\theta_{2}^{\prime }\mathrm{tr}\left(H_{2}\Lambda \left(\phi \right)\right)\right\},\ldots ,ℜ\left\{\theta_{N}^{\prime }\mathrm{tr}\left(H_{N}\Lambda \left(\phi \right)\right)\right\}\right]}^{T}. \end{array}$$

**Remark** **2.** *The gradient is computed at $O(MNK+M^{2}K+M^{3})$ with complexity O(MNK) for ${\left\{\mathrm{tr}\left(H_{n}\Lambda \left(\phi \right)\right)\right\}}_{n=1}^{N}$, $O(MNK+M^{2}K+M^{3})$ for $\Lambda \left(\phi \right)=\Xi H^{H}{\tilde{\Theta }}^{H}Y^{-1}\left(\phi \right)$, O(MNK) for $X_{1}=\Xi H^{H}{\tilde{\Theta }}^{H}$, $O(M^{2}K)$ for $Y\left(\phi \right)=I_{M}+\tilde{\Theta }HX_{1}$, $O(M^{3})$ for $Y^{-1}\left(\phi \right)$, and $O(M^{2}K)$ for $\Lambda \left(\phi \right)=X_{1}^{H}Y^{-1}\left(\phi \right)$. In comparison, numerical differentiation for $\frac{\partial R(\phi )}{\partial \phi_{n}}$ requires $O(M^{3}N+M^{2}KN+MKN^{2})$ since $R\left(\phi \right)=\log_{2}\det\left(Y\left(\phi \right)\right)$ is computed for each element at $O(M^{3}+M^{2}K+MNK)$.*

**Remark** **3.** *The complexity of the BFGS, L-BFGS, and GD comprising only N-dependent terms is given by $O(\left(MNK+N^{2}\right)I_{bfgs})$, $O(\left(MNK+m_{B}N\right)I_{lbfgs})$, and $O(\left(MNK+N\right)I_{gd})$ with $I_{\chi }$ iterations until a convergence for each method χ. The sequential optimization in [7] requires $O(\left(M^{3}N+MKN\right)I_{conv})$ with $I_{conv}$ iterations in updating ϕ since a matrix inversion and an eigenvalue decomposition of complexity $O(M^{3}+MK)$ are required for each element.*

## 4. Simulation Results

**Figure 2:**
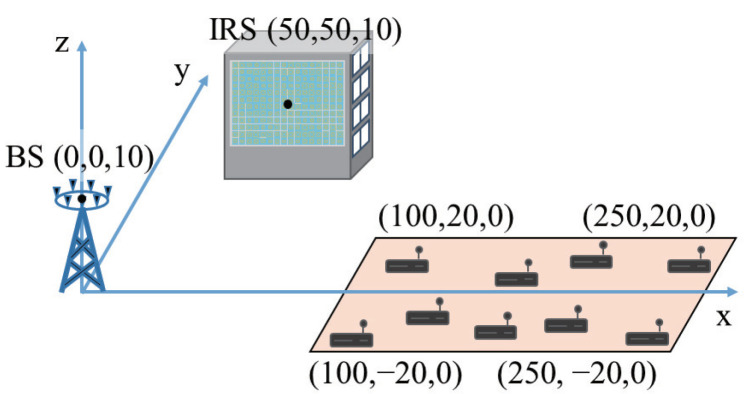
Simulation setup for the IRS-aided uplink NOMA.

The performance of the IRS-aided uplink NOMA is evaluated when the maximum transmit power is set to $P_{k}^{\max}=23$ dBm for k∈K and the noise power is set to $\sigma^{2}=-100$ dBm. Practical IRS reflection is modeled with α=1.6, $\beta_{\min}=0.2$, and $\phi_{0}=0.43\pi$ as in Ref. [32]. We set the tolerance of the algorithms to $10^{-5}$ up to maximum 500 iterations. The simulation setup is illustrated in Figure 2, where the (x,y,z) coordinates are given in meter. The BS and IRS are located at (0,0,10) and (50,50,10), respectively, whilst the devices are uniformly distributed in the shaded rectangular region bounded by (100,−20,0), (100,20,0), (250,−20,0), and (250,20,0).

The channels are modelled as

(37) $$\begin{array}{ccc} & & \;G=\sqrt{\frac{\kappa_{R,B}\omega_{R,B}}{\kappa_{R,B}+1}}G_{\mathrm{LoS}}+\sqrt{\frac{\omega_{R,B}}{\kappa_{R,B}+1}}G_{\mathrm{NLoS}}, \end{array}$$

(38) $$\begin{array}{ccc} & & \;f_{k}=\sqrt{\frac{\kappa_{k,R}\omega_{k,R}}{\kappa_{k,R+1}}}f_{\mathrm{LoS},k}+\sqrt{\frac{\omega_{k,R}}{\kappa_{k,R+1}}}f_{\mathrm{NLoS},k},\;k\in K, \end{array}$$

(39) $$\begin{array}{ccc} & & \;v_{k}=\sqrt{\frac{\kappa_{k,B}\omega_{k,B}}{\kappa_{k,B+1}}}v_{\mathrm{LoS},k}+\sqrt{\frac{\omega_{k,B}}{\kappa_{k,B}+1}}v_{\mathrm{NLoS},k},\;k\in K, \end{array}$$

where the subscripts LoS and NLoS represent the line-of-sight (LoS) and non-LoS (NLoS) components, respectively, and $\omega_{x,y}$ and $\kappa_{x,y}$ denote the path loss and Rician factor between nodes *x* and *y* for x,y∈K∪{R,B} with R for the IRS and B for the BS. The path loss is given by $\omega_{x,y}=10^{-3}d_{x,y}^{-\nu_{x,y}}$ at distance $d_{x,y}$ with path loss exponent $\nu_{x,y}$, where $\nu_{R,B}=2.2$, $\nu_{k,R}=2.8$, and $\nu_{k,B}=4$. The Rician factor is set to $\kappa_{R,B}=\kappa_{k,R}=2$ and $\kappa_{k,B}=0$. We model the LoS components with a uniform linear array for the BS and an $N_{v}\times N_{h}$ uniform planar array constructed by $N_{v}=8$ and $N_{h}=N/8$ for the IRS as [7]

(40) $$\begin{array}{ccc} & & \;G_{\mathrm{LoS}}=a_{B}\left(\varphi_{R,B}^{A}\right)a_{R}^{H}\left(\varphi_{R,B}^{D},\vartheta_{R,B}^{D}\right), \end{array}$$

(41) $$\begin{array}{ccc} & & \;f_{\mathrm{LoS},k}=a_{R}\left(\varphi_{k,R}^{A},\vartheta_{k,R}^{A}\right),\;k\in K \end{array}$$

where $a_{B}\left(\varphi \right)\in C^{M}$ and $a_{R}\left(\varphi ,\vartheta \right)\in C^{N}$ are the array response at the BS and IRS, respectively, defined in [7] with the azimuth (elevation) angle-of-arrival $\varphi_{x,y}^{A}$ ($\vartheta_{x,y}^{A}$) and angle-of-departure $\varphi_{x,y}^{D}$ ($\vartheta_{x,y}^{D}$) from node *x* to node *y*. Specifically, for the antenna and IRS with half-wavelength element spacing, we have

(42) $$\begin{array}{c} a_{B}\left(\varphi \right)={\left[1,e^{j\pi \sin(\varphi )},\ldots ,e^{j\pi (M-1)\sin(\varphi )}\right]}^{T} \end{array}$$

and

(43) $$\begin{array}{c} {\left[a_{R}\left(\varphi ,\vartheta \right)\right]}_{n}=e^{j\pi (\left\lfloor \frac{n}{N_{v}}\right\rfloor \sin\vartheta \sin\varphi +\left(n-N_{v}\left\lfloor \frac{n}{N_{v}}\;\right\rfloor \right)\sin\vartheta \cos\varphi )} \end{array}$$

for φ∈[0,2π) and ϑ∈[−π/2,π/2). The NLoS components are modeled to be uncorrelated complex Gaussian.

**Figure 3:**
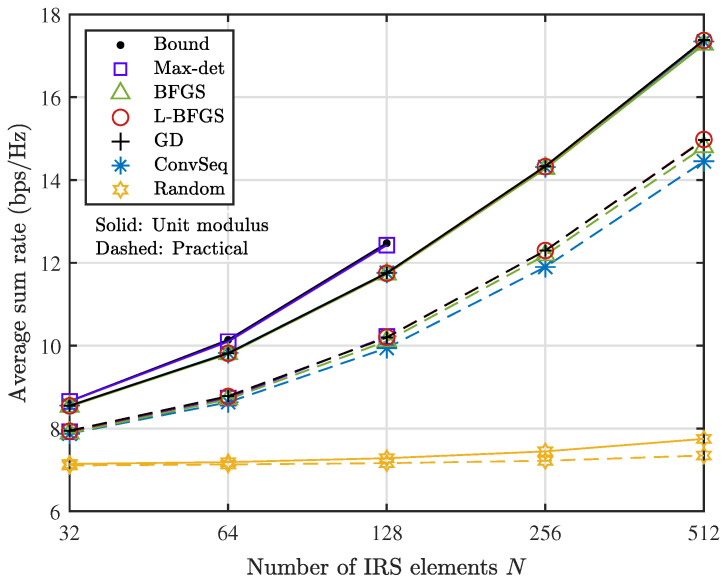
Average sum rate as the number *N* of IRS elements increases when M=2 and K=4.

The average sum rate of the network is shown as the number *N* of IRS elements increases in Figure 3 when M=2 and K=4. We provide the results with unit modulus reflection and practical reflection drawn with solid and dashed lines, respectively. Bound represents the upper bound ${\tilde{R}}_{⋄}=\tilde{R}\left({\tilde{X}}_{⋄}\right)$ obtained with the solution ${\tilde{X}}_{⋄}$ of (22), and Max-det denotes the performance of a feasible solution $\phi_{l_{☆}}$ derived from ${\tilde{X}}_{⋄}$ through Gaussian randomization with L=50. Bound and Max-det are shown up to N=128 due to the formidable computational time in solving (22) for a large *N*. BFGS, L-BFGS with $m_{B}=10$, and GD implemented with the derived gradient are compared with ConvSeq denoting the sequential optimization in Ref. [7]. The results with random IRS phases denoted by Random are also added to serve as a lower bound. Clearly, the average sum rate increases steeply with *N* by optimizing the IRS reflection. In cases of unit modulus reflection, Max-det provides the best performance close to the optimal one estimated by Bound for a moderate *N*. The iterative algorithms provide almost the same performance under unit modulus reflection, but BFGS, L-BFGS, and GD provide a slight gain over ConvSeq increasing with *N* under practical reflection.

**Figure 4:**
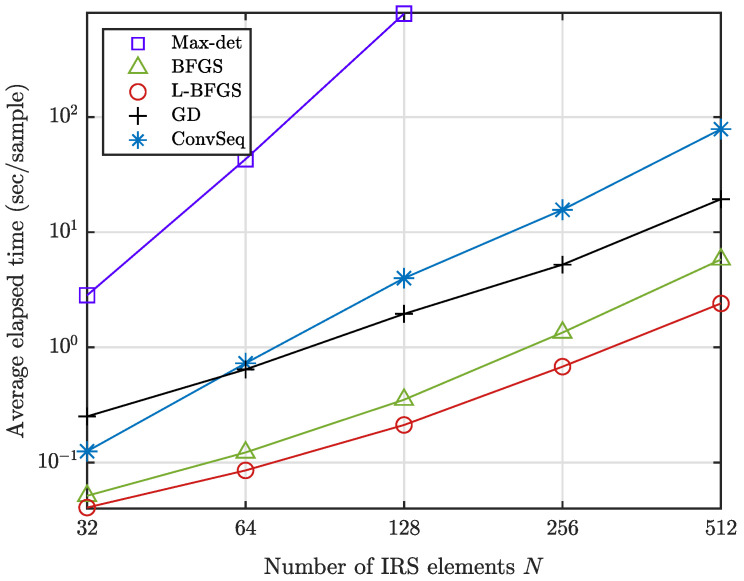
Computational time of unit modulus reflection as the number *N* of IRS elements increases when M=2 and K=4.

However, the gradient-based iterative algorithms are observed to reduce the computational time, as shown in Figure 4, which provides the average evaluation time per sample in obtaining the results of unit modulus reflection in Figure 3 with Intel(R) Xeon(R) Gold 6226R CPU @ 2.90Hz. Clearly, the iterative algorithms exhibit a significant reduction in computational time over Max-det at the cost of a performance loss. The computational time of GD is comparable to that of ConvSeq since both GD and ConvSeq require a large number of iterations until their convergence. L-BFGS provides the best computational time, with less complexity in updating the solution than BFGS and with a smaller number of iterations than GD by finding a better search point through inverse Hessian estimation. Thus, L-BFGS with the derived gradient would be a choice of practical merit for a large *N*.

**Figure 5:**
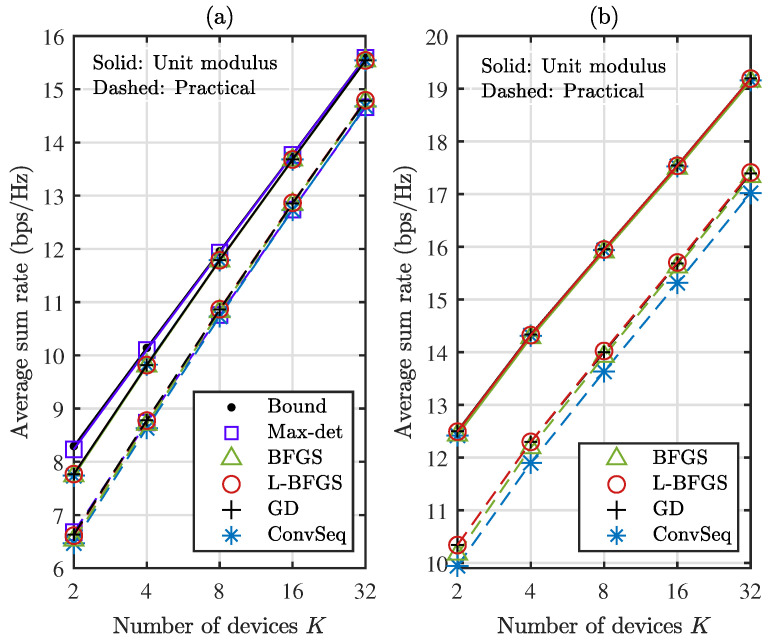
Average sum rate as the number *K* of devices increases when M=2 and K=4: (**a**) N=64 (**b**) N=256.

**Figure 6:**
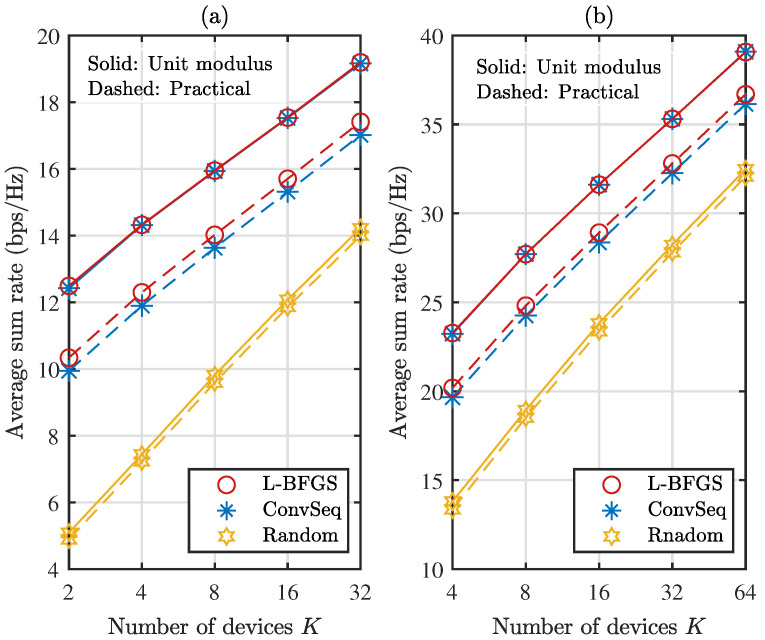
Average sum rate as the number *K* of devices increases when N=256: (**a**) M=2 (**b**) M=4.

Figure 5 compares the average sum rate as the number *K* of devices increases when M=2, with N=64 in Figure 5a and N=256 in Figure 5b. The average sum rate increases as the number *K* of devices increases. In addition, the gradient-based methods and ConvSeq provide a similar performance except for practical reflection with N=256. When N=64 in Figure 5a, the performance of the gradient-based algorithms becomes close to the optimal one as *K* increases for unit modulus reflection. When N=256 in Figure 5b, the gradient-based algorithms outperform ConvSeq by about 0.3 dB for practical reflection. Again, L-BFGS provides a good performance among the gradient-based algorithms. Hence, we compare the performance of L-BFGS and ConvSeq for different numbers *M* of antennas with N=256 in Figure 6; we set M=2 in Figure 6a and M=4 in Figure 6b. The sum rate becomes almost doubled by doubling the number of antennas. Again, L-BFGS provides a similar or slightly improved performance compared with ConvSeq, which is obtained at a computational time about 11% and 18% of ConvSeq with M=2 and 4, respectively, for most *K* values in the figures.

Discussion: It is noteworthy that the nonlinear optimization problem in (24) with respect to the IRS phase vector ϕ is a non-convex optimization problem that exhibits multiple local minima in general. Hence, the iterative algorithms considered herein do not guarantee a convergence to the global optimal point, but to one of the local minima. The gradient-based algorithms updating *N* variables simultaneously for the next search point tend to find a similar local minimum at a different convergence rate. However, ConvSeq, updating one variable at a time, tends to find a worse point in particular for practical reflection since it resorts to limited information for the next search point. To improve the performance, we may run an iterative algorithm with different initial points, resulting in different local minima so that a better solution can be found. However, it is observed that the gain is trivial for this problem. From this, devising a new algorithm filling the gap to the optimal performance with a complexity between those of Max-det and gradient-based algorithms would be an interesting topic for further study.

## 5. Concluding Remarks

We have considered a sum rate maximization problem for the IRS-aided uplink multiantenna NOMA under a generalized reflection model including unit and phase-dependent amplitudes. We have solved the problem through extended SDR to obtain an upper bound on the sum rate and a near-optimal solution for a moderate-sized IRS. We have applied the gradient-based iterative algorithms for a large-sized IRS by providing the gradient in an explicit form under generalized reflection. The results show that, among the gradient-based algorithms, L-BFGS implemented with the derived gradient provides a more competitive solution than the conventional method in both computational time and performance.
